# Animal Silk-Derived Amorphous Carbon Fibers for Electricity Generation and Solar Steam Evaporation

**DOI:** 10.3389/fchem.2021.669797

**Published:** 2021-06-22

**Authors:** Ping Qi, Jing Ren, Shengjie Ling

**Affiliations:** School of Physical Science and Technology, ShanghaiTech University, Shanghai, China

**Keywords:** silk, carbonization, evaporation, electricity, fabric

## Abstract

Animal silk-derived carbon materials are of interest to various applications, such as smart cloth and wearable sensors. However, it remains a challenge to massively transform silks into continuous carbon fibers. In this work, carbon fibers based on two kinds of animal silks, i.e., *Bombyx mori* (*B. mori*) silk and *Antheraea pernyi* (*A. pernyi*) silk, are prepared by using a large-scale-capable one-step heating process without any additives or activation process. These carbon fibers and yarns are electroconductive and mechanically robust. To expand the application of these carbonized silks, we further weaved them with cotton yarns to obtain composite fabrics with different textures and evaluated their performance for solar steam evaporation. Our results confirmed that the advantages of these composite fabrics in light absorption, large surface area, and hierarchical liquid transport channels allowed them to be used as a solar steam generation for desalination and sewage treatment. In addition, we reported that these conductive carbon fibers could be assembled into fluidic nanogenerators to generate electricity from the water flow. This work is expected to guide a large-scale preparation and use of animal silk-derived amorphous carbon fibers.

## Introduction

Carbon fibers can be produced from animal silks through a high-temperature carbonization as the fiber morphology of silks can be maintained after the carbonization (Khan et al., 2007; Cho et al., 2015, 2017). A high crystalline content, i.e., a high β-sheet content, is believed to play an essential role in maintaining the structural stability of a carbonized silk because the β-sheet structure can change into a multi-hexagonal carbon structure—a heat-resistant structure to withstand a higher carbonization temperature (Cho et al., 2015). Indeed, the β-sheet content of *Bombyx mori* (*B. mori*) silk, one of the most common cocoon silks used for carbonization, is as high as 50%. However, spider dragline silk with a β-sheet content of only about 15–20% has also been proved to retain the fiber morphology well after the carbonization (Ling et al., 2011; Paquet-Mercier et al., 2013). Therefore, there is no doubt that other structures, such as molecular chain orientation, nanofibrils, and their associated interfaces, in animal silks should also play an essential role in the structural stability and the mechanical performance of a carbonized silk. A detailed carbonization mechanism of animal silks in this regard deserves further analysis (Majibur Rahman Khan et al., 2009; Cho et al., 2015, 2017). Comparing the carbonized structures of different silks will undoubtedly promote understanding of the structure–property relationship of a carbonized silk family.

Another significance of silk carbonization is to provide a new route to generate carbon fibers (Khan et al., 2007; Cho et al., 2017; Cao et al., 2018). With a series of environmental issues caused by the petrochemical industry, the production and use of synthetic polymer fibers have been increasingly restricted. This situation forces the scientific and industrial communities to look for carbon fiber precursors from the nature instead of relying on synthetic polymer fibers, such as polyacrylonitrile (PAN). Indeed, in these years, carbon materials from natural sources have been widely reported (Fu et al., 2017; Jeon et al., 2017; Xu N. et al., 2017; Wang et al., 2019; Liang et al., 2020). Apart from the advantages of sustainability, the advantages of nature-sourced carbon fibers in the mechanical and electric properties have also been noticed (Wang et al., 2017, 2019; Xu N. et al., 2017). Most of these carbon fibers have a decent mechanical strength for mechanized processing, such as knitting and weaving while maintaining flexibility for arbitrary deformation. These merits allow a complex textile structure design and further decoration to achieve a desired porous morphology for different applications, such as flexible energy devices and wearable electronics (Xu et al., 2018; Zheng et al., 2020).

In this work, a large-scale-capable one-step heating process without any additives or activation process is explored to produce carbon fibers from two animal silk products, *B. mori* silk and *Antheraea pernyi* (*A. pernyi*) silk. The resulted carbonized silks maintained their original morphology while they were mechanically robust and electrically conductive. These merits allow these carbonized silks to be co-weaved with other yarns, such as cotton yarns, into functional fabrics. We then explored the applications of these carbonized silk fabrics in fluidic fiber generators and solar steam generations.

## Experiment

### Carbonization of Silk Yarns

*Bombyx mori* silk and *A. pernyi* silk were purchased from Suzhou Huguang silk company, Jiangsu province, China. Commercial cotton yarns were purchased from Shanghai Yasuwang company, Shanghai, China. Before the carbonization, silks were arranged into yarns, which were composed of 20 filaments, and then arranged into bundles, which were obtained by the decomposition of 20 yarns. The silk bundles were carbonized in a high-temperature tube furnace (Shanghai Jvjing Precision Instrument Manufacturing Co., Ltd., Shanghai, China). During a one-step carbonization process, silk bundles composed of yarns were directly heated to a desired temperature (800, 1,000, and 1,250°C) in nitrogen atmosphere at a heating rate of 5°C/min. Then, the samples were maintained in a desired temperature for 30 min and were cooled to room temperature in nitrogen atmosphere. For practical use, the carbonized silk bundles were twisted into yarns by using a custom yarn-spinning device.

### Characterization

For the tensile tests, every filament was fixed on an Instron 5966 machine (Instron, Norwood, MA, USA). The strain rate was set at 2 mm/min. The morphology of fibers was observed by high-resolution scanning electron microscopy (SEM; JEOL JSM7800F, Tokyo, Japan) at an acceleration voltage of 5 kV. The structure of carbonized fibers was detected by x-ray diffraction (XRD; Bruker, D8). A custom-made micro-Raman system with ANDOR SR500i-D2-R spectrometer and 1,800 grooves per mm grating was used. The incident laser wavelength was 532 nm. Wide-angle XRD was carried out at the Characterization and Analysis Center of ShanghaiTech University by using the Xenocs WAXS equipment, Xeuss 2.0. The diffraction patterns were collected by a detector with 619 pixels × 487 pixels of each area being 172 × 172 μm. The wavelength and the photon flux of the x-ray source were 1.54189 Å and 4.0 × 10^7^ photons s^−1^, respectively. The beam size was 1.2 × 1.2 mm. The resistance of a fiber was recorded by a multichannel touchscreen digital meter (Tektronix DMM6500, Beaverton, OR, USA). The electrical conductivity of a fiber was calculated by the resistance and the average diameter.

### Measurements for Fluidic Fiber Nanogenerator

Copper wires were used to connect the two electrodes [a 4-cm carbonized *B. mori* silk (CBS) yarn and a 4 cm copper wire] with an external circuit: an electrochemical workstation (CHI 660, CH Instruments Inc., Shanghai, China). Polydimethylsiloxane (PDMS) was adopted to seal the conducting wires and the connection parts to avoid electric leakage. Before testing, aqueous KCl solution (0.6 M) was placed under a fiber nanogenerator, which was controlled by a motor to mimic the motion of ocean waves and create an infiltration or a separation between solution and the nanogenerator (Xu N. et al., 2017). During the test, an electrochemical workstation monitored the output voltage generated by a fluidic fiber nanogenerator with a solvent motion velocity of 0.78 cm/s.

### Solar Stem Generation Performance Evaluation

Rhodamine-B, sodium chloride, and vegetable oil were purchased from Aladdin Biochemical Technology Co., Ltd., Shanghai, China. An AM 1.5 solar simulator (SAN-EIELECTRIC, XES-40S3-TT, Osaka, Japan) was used as a light source to carry out the steam generation evaluation experiments at 24°C room temperature and ~50% humidity. The mass changes of salt solution, oil-in-water emulsion, and dyed Rhodamine-B solution were recorded by a precision balance to calculate the evaporation rate (*m*) and conversion efficiency (η) according to Equations (1) and (2), respectively:

(1) $$\begin{array}{l} m=\Delta m\times 10/A \end{array}$$

(2) $$\begin{array}{l} \eta =m^{\prime }\times \;\left(L_{V}+Q\right)/P_{in} \end{array}$$

where *m* (kg m^−2^ h^−1^) is the evaporation rate, Δ*m* (g) is the mass change in 1 h, *A* is the area of fabrics, η (%) is the efficiency of solar to steam, and *m*′ is the evaporation rate after the subtraction of the evaporation rate under dark conditions. *L*_*V*_ is the latent heat of vaporization of water $\left(L_{V}\left(T\right)=1.91846\times 10^{6}{\left[T/\left(T-33.91\right)\right]}^{2}{\text{ J kg}}^{-1}\right)$, where *T* is the temperature of vaporization; and *Q* is the sensible heat of water of unit mass $\left(Q=c\left(T_{2}-T_{1}\right){\text{ J kg}}^{-1}\right)$, where *c* is the specific heat of water, which can be assumed as constant (4.2 J g^−1^ K^−1^), *T*_2_ is the temperature of vaporization, and *T*_1_ is the initial temperature of water, and *P*_in_ is the incident solar power on the device surface (Henderson-Sellers, 1984; Li et al., 2019).

### Water Purification

The stabilized oil-in-water emulsion (200 ppm) was prepared by mixing 0.05 g of vegetable oil with 249.95 g of deionized water and stirred for 10 min. Dyed solution (200 ppm) was obtained by intermixing 0.05 g of Rhodamine-B with 249.95 g of deionized water. About 8.75 g of KCl was added into 241.25 g of deionized water to obtain the simulated seawater with 3.5 wt%. Water purification was performed by collecting the vapor generated from these solutions (simulated seawater, dyed Rhodamine-B solution, and oil-in-water emulsion) by the CBS/cotton solar steam generators. Before testing, a CBS/cotton fabric was put on a column polystyrene (PS) foam, which was then floated on the solutions for at least 10 min to reach to a stable state. Then, the CBS/cotton solar steam generator was put on a balance and exposed to one sun illumination for 60 min. During this process, the mass was recorded for every 5 min.

## Results and Discussion

**Figure 1:**
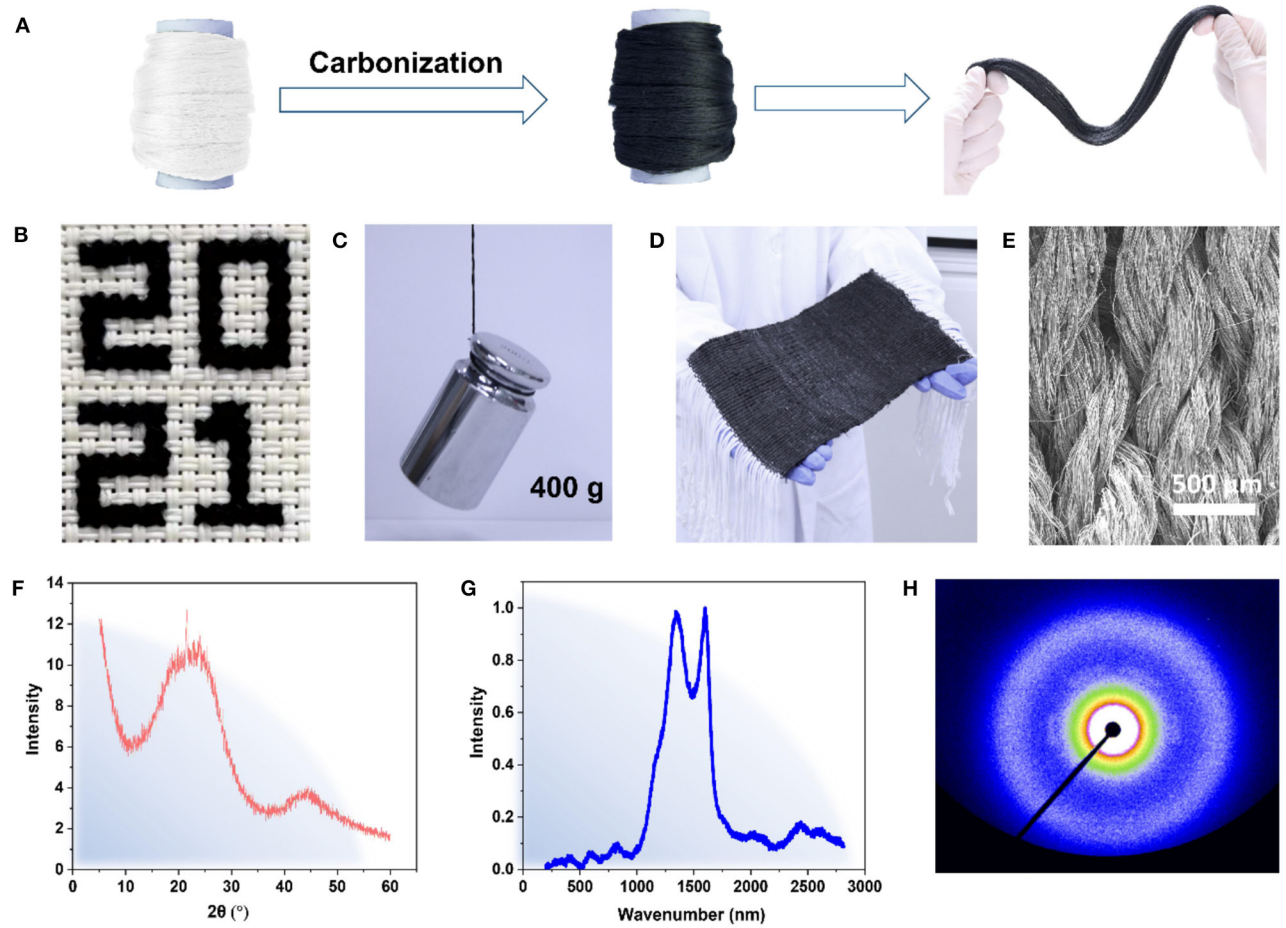
**(A)** The morphology of *Bombyx mori* (*B. mori*) silks before and after the carbonization; **(B)** “2021” is knitted by carbonized *B. mori* silks (CBSs); **(C)** A weight of 400 g is lifted by CBSs (0.1 g); **(D)** A large-scale fabric is made by CBSs and cotton yarns; **(E)** The scanning electron microscopy (SEM) image of a composite fabric; **(F)** x-ray diffraction (XRD) spectrum of CBSs at carbonization temperature of 1,000°C; **(G)** Raman spectra of CBSs at carbonization temperature of 1,000°C; and **(H)** Wide-angle scattering diffraction pattern of CBSs at carbonization temperature of 1,000°C.

The carbonization of *B. mori* silks can be conducted at different temperatures more than 800°C. In this study, three heating temperatures, i.e., 800, 1,000, and 1,250°C, were chosen as these temperatures can be achieved with conventional furnace equipment, instead of relying on expensive heat treatment equipment. Figure 1A compares the morphology of *B. mori* silks before and after the carbonization. It can be found that the *B. mori* silks retain the fiber morphology after the carbonization, but all become black. The resultant CBSs are mechanically robust and flexible, are easy to handle, and can tolerate regular machining operation (Figure 1B). It can easily lift an object that exceeds 4,000 times its own weight (Figure 1C), can be twisted into yarns by a customized yarn-spinning device **(**Movie 1), and also can be knitted with cotton yarns into a piece of composite fabric (Figures 1D,E). For a case, the results from both XRD (two broad peaks around 2θ = 24° and 2θ = 45°; Wang et al., 2015; Wu et al., 2017) and Raman spectroscopy (the characteristic bands of D band at 1,335 cm^−1^ and G band at 1,587 cm^−1^; Bukalov et al., 2018; Zhang et al., 2020) indicate that the carbonized silks are composed of amorphous carbon (Figures 1F,G). For another case, a negligible azimuthal intensity anisotropy of wide-angle x-ray two dimensional (2D) scattering patterns reveals an isotropic carbon arrangement in the CBSs (Figure 1H; Cho et al., 2017).

For comparison, we also carbonized the fibers of PAN and polyamide 66 (PA66) under the same experimental conditions. However, both fibers shrank and melted dramatically (Supplementary Figure 1). This is a surprising result because both PAN and PA66 fibers are the most commonly used precursors for making carbon fibers. These two synthetic fibers cannot maintain the structural stability in a one-step carbonization process, which can be explained by the lack of pre-oxidation. The pre-oxidation has been shown to be critical for the formation of a synthetic polymer-derived carbon fiber (Zhu et al., 2013; Cao et al., 2018). During the pre-oxidation, the chain molecules in the synthetic polymer can gradually change into a heat-resistant trapezoidal structure to withstand a higher carbonization temperature (Zhao et al., 2016; Elagib et al., 2018).

**Figure 2:**
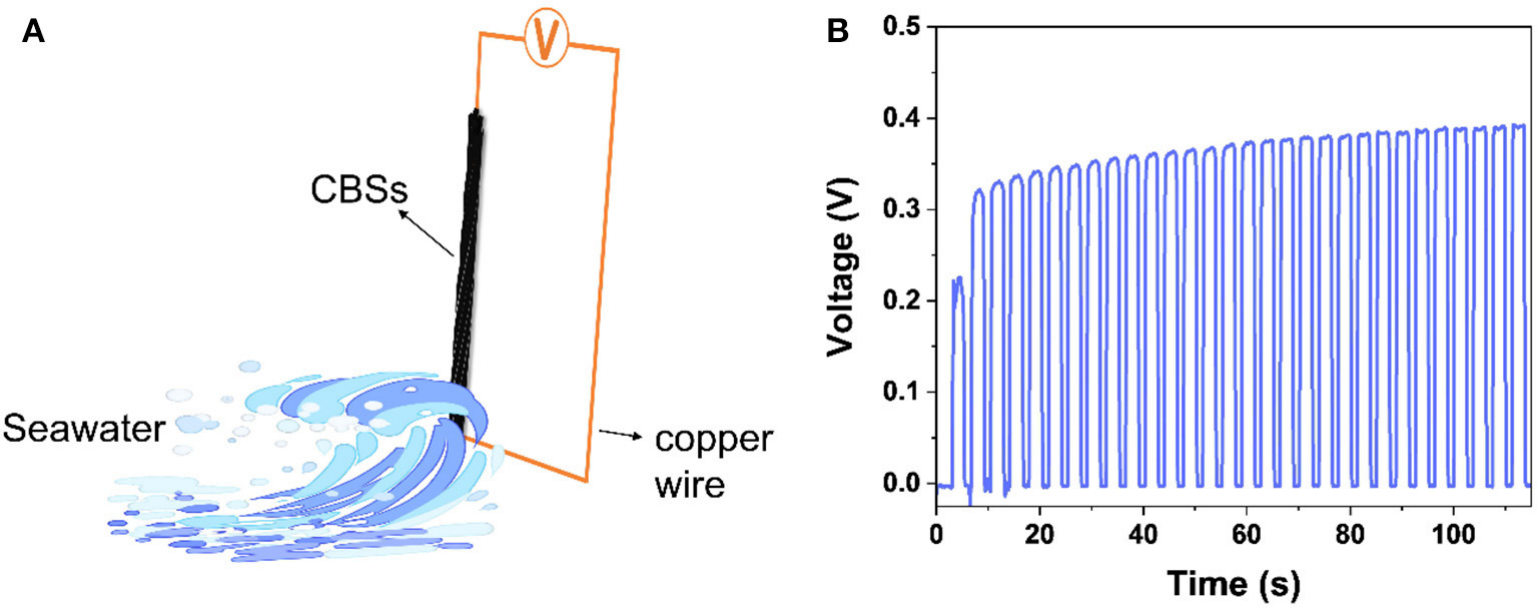
**(A)** The scheme of the CBS generator for electricity generation; and **(B)** Electricity generated by a generator (4 cm copper electrode and 4-cm carbon electrode) while it moved in KCl solution of 0.6 M heated to 42°C at a velocity of 0.78 cm/s.

As summarized in Supplementary Table 1, CBSs are electroconductive, with electrical conductivity changing from 18 to 2,387 S cm^−1^, depending on the carbonization temperature. It is worth mentioning that the conductivity of the CBSs or their fabric is comparable to that of the crystallized carbon fibers (10^2^–10^4^ S cm^−1^; Zhang et al., 2000; Newcomb, 2016) and carbon nanotube yarns (10^3^–10^4^ S cm^−1^; Lekawa Raus et al., 2014; Chinnappan et al., 2018). Therefore, the CBS shows potential applications in devices requiring conductivity. Herein, a CBS-based fluidic electrogenerator was assembled to generate electricity from the water flow (Figure 2). Benefiting from the aligned but rough surface morphology of CBS yarns (Supplementary Figure 2), they can be applied directly as a flexible electrode with a copper wire as the other electrode. Electricity was derived from the relative movement between the CBS electrode and the solution and then transferred to an external circuit (Figure 2A). In a primary model, the output voltage of 0.2 V was generated by a generator assembled by a 4 cm copper wire electrode and a 4-cm carbonized CBS electrode while moving in 0.6 M KCl aqueous solution at a velocity of 0.78 cm/s. The output voltage further increased to 0.35 V when the saline solution temperature raised to 42°C (Figure 2B), which is an ordinary temperature for the sea under the sun. The output voltage is positively correlated with the effective solution contact area of the yarn electrode (Xu N. et al., 2017). The hydrophobic property and porous morphology of the CBS yarn with a large amount of absorbed air resulted in an inefficient solution/electrode contact. This phenomenon can be alleviated with the repeated flow/backflow of the solution, and then the output voltage will increase accordingly. The output voltage of the devices can further increased by increasing the immersing yarn length and by the surface modification of electrode to increase surface area or improve hydrophilicity.

To quantitatively evaluate the mechanical properties of the CBSs, their tensile stress-strain curves were measured. The tensile mechanical performances of the carbonized *A. pernyi* silks (CASs) were also tested for comparison. Their stress-strain curves are presented in Supplementary Figure 3, and their corresponding mechanical properties are summarized in Supplementary Table 2. It can be found that the Young's modulus and strength of the carbonized silks increased by an increase in the heating temperature. For example, the carbonization of the CBSs at a temperature of 1,250°C maintained a decent toughness of 26 ± 20 MJ/m^3^ with a strength of 30 ± 13 MPa and a Young's modulus of 1.5 ± 0.6 GPa. Such a mechanical performance is sufficient to ensure that these carbonized silks can be used for subsequent processing. By contrast, a tensile strength and Young's modulus of the CBSs are around 2–3 times higher than that of the CASs with the same carbonization temperature under the same condition. Our tentative experiments also proved that the CASs could not be twisted, knitted, and weaved.

**Figure 3:**
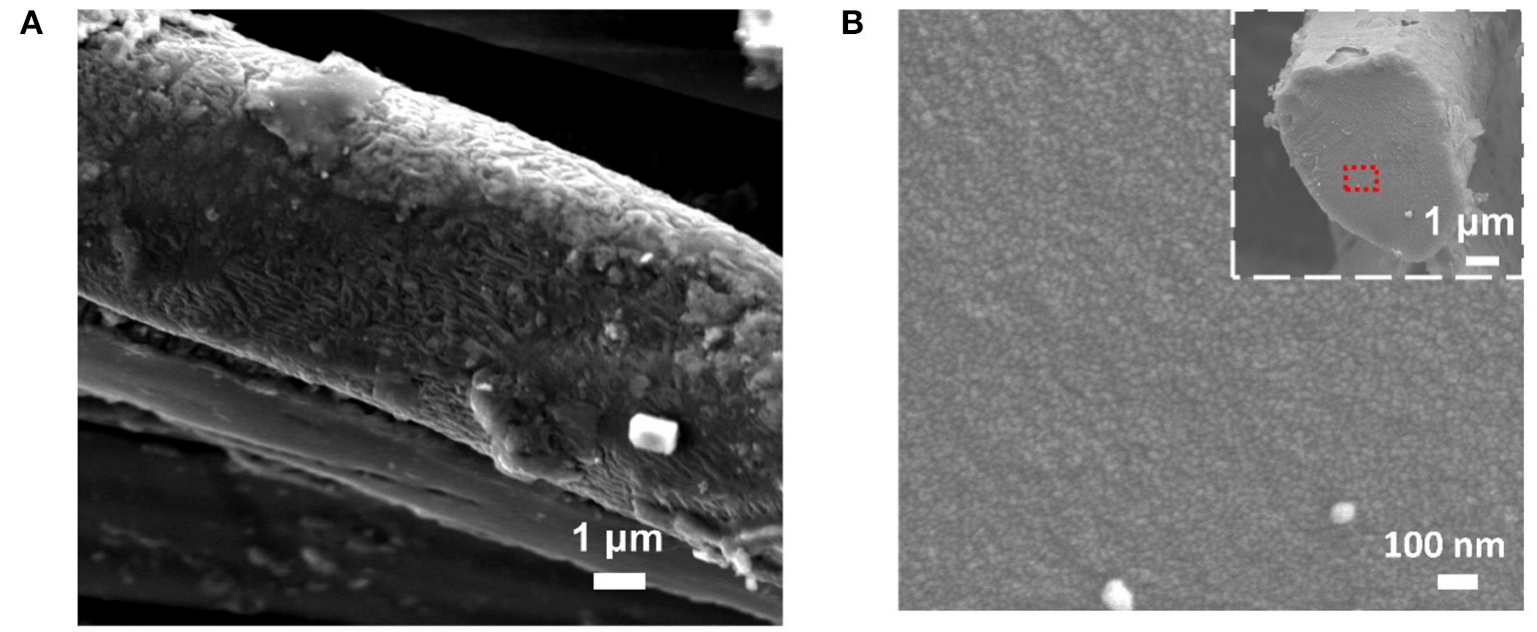
**(A)** SEM image of CBSs with the carbonization temperature of 1,000°C; and **(B)** Cross section image of CBSs and zoomed cross section image of CBSs with the carbonization temperature of 1,000°C.

Scanning electron microscopy was used to examine the mesostructure of the CBS and CAS. As shown in Supplementary Figure 2, an intact fiber morphology of the CBSs was maintained even after a twisting process. Wrinkle structures were detected on the surface of the single carbonized silk fibers (Figure 3A), which should be caused by uneven radial shrinkage during the heating. Free volumes in the amorphous region contribute more to the shrinkage under high temperature while the β-sheet structure is more compact and regular, then the shrinkage of β-sheet structure is low during the carbonization (Cho et al., 2015). The cross-sectional SEM image (Figure 3B) clearly reveals that the cross section of the single CBS is dense and uniform without detectable defects and voids. In contrast, many voids with a diameter of around 0.044–0.125 μm were observed in the cross section of the CAS (Supplementary Figure 4 and Supplementary Table 3). This observation agrees with the structural difference of natural *B. mori* and *A. pernyi* silks in a mesoscale. Compared with *B. mori* silks, *A. pernyi* silk presents a more obvious defect structure. The defects (e.g., cavities, cracks, surfaces, and tears) with a width around several to hundreds of nanometers are often detected in *A. pernyi* silk, which are mainly evolved from vacuoles in silk spinners, and its volume percentage can be as high as 30–50% (Zhang Q. et al., 2018; Lin et al., 2019). Meanwhile, *B. mori* silks are dense in such a mesoscale with tightly packed nanofibrils. The cavities in *A. pernyi* silk might fuse at high temperature, but some obvious defects were retained after the carbonization.

Interestingly, the impact of these defects on the mechanical properties of the silk is usually positive (Lin et al., 2019). These defects can improve the ductility and damage tolerance of fibers through restricted fibril shearing, controlled slippage, and cleavage (Lin et al., 2019). However, in terms of carbon fiber, the impact of these defects on its mechanical properties is catastrophic because the defects in an inorganic material are seeds for the fracture failure due to the localized stress concentrations (Wu et al., 2016). Accordingly, the mechanical properties of the CAS are much weaker than those of the CBS.

**Figure 4:**
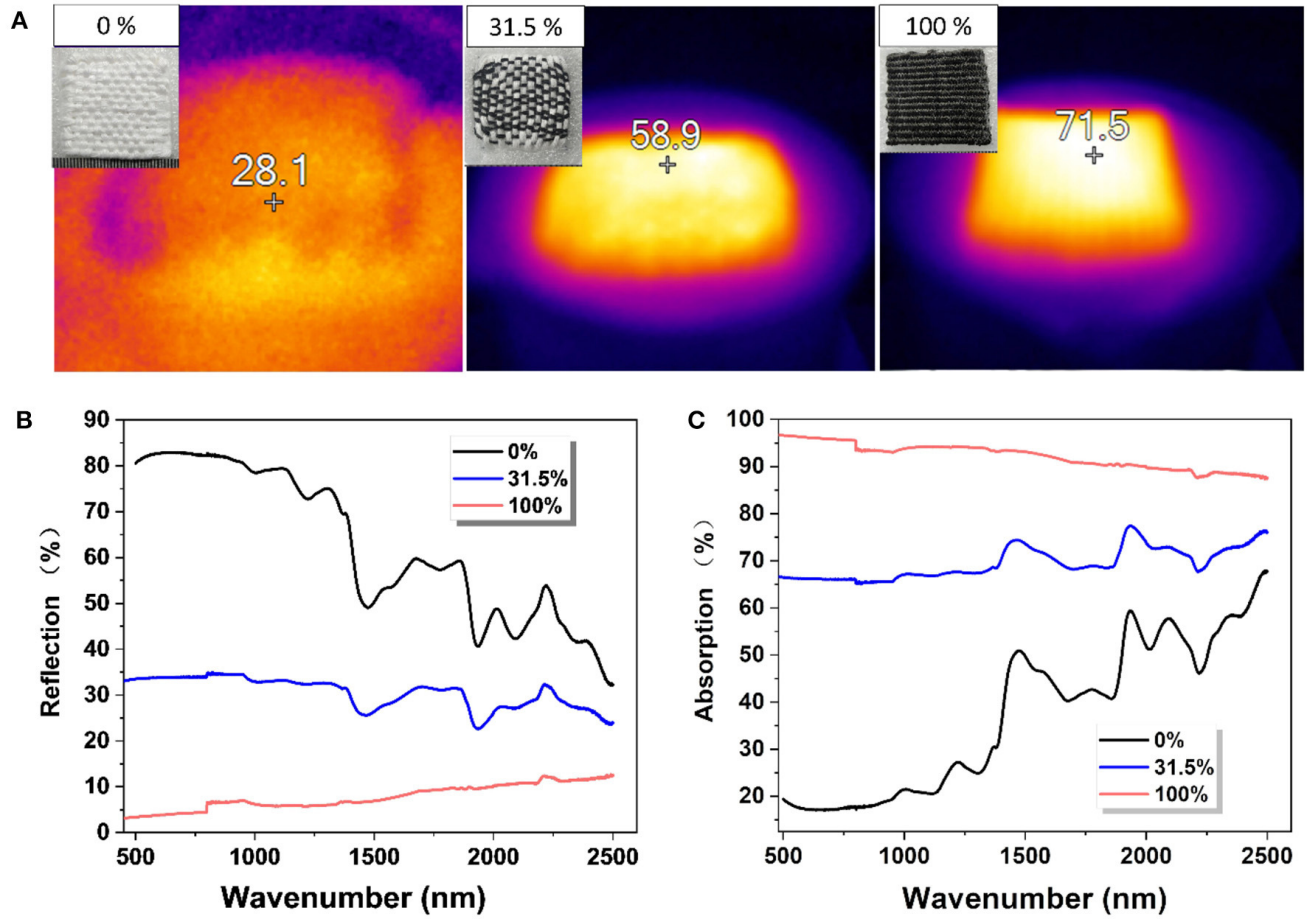
**(A)** The steady surface temperature of fabric generators (4 × 4 cm) with different values of CBS coverage under one sun illumination; **(B)** The reflection under increased wavenumbers for fabric generators with different values of CBS coverage; and **(C)** The absorption under increased wavenumbers for fabric generators with different values of CBS coverage.

As the CBSs are mechanically robust and black in color, we weaved them with cotton yarns into the fabric-based solar steam generators and tested their photothermal conversion efficiency (Zhang Q. et al., 2018; Li et al., 2019). Figure 4 shows the heat conversion, reflection of sunlight, and absorption of sunlight of three plain-weaving CBS/cotton fabrics with different exposed surface areas of the CBS yarns (0, 31.5, and 100%, respectively, calculated by Image J software). When the exposed area of CBS yarns increased from 0 to 31.5%, the surface temperature significantly increased from 28.1 to 58.9°C. The surface temperature of the generator even reached up to 71.5°C when the CBS yarns were at full coverage (Figure 4A). Not surprisingly, the solar steam generator with 100% CBS coverage showed the lowest reflection and highest absorption across the full spectrum with a wavenumber ranging from 500 to 2,500 nm (Figures 4B,C).

**Figure 5:**
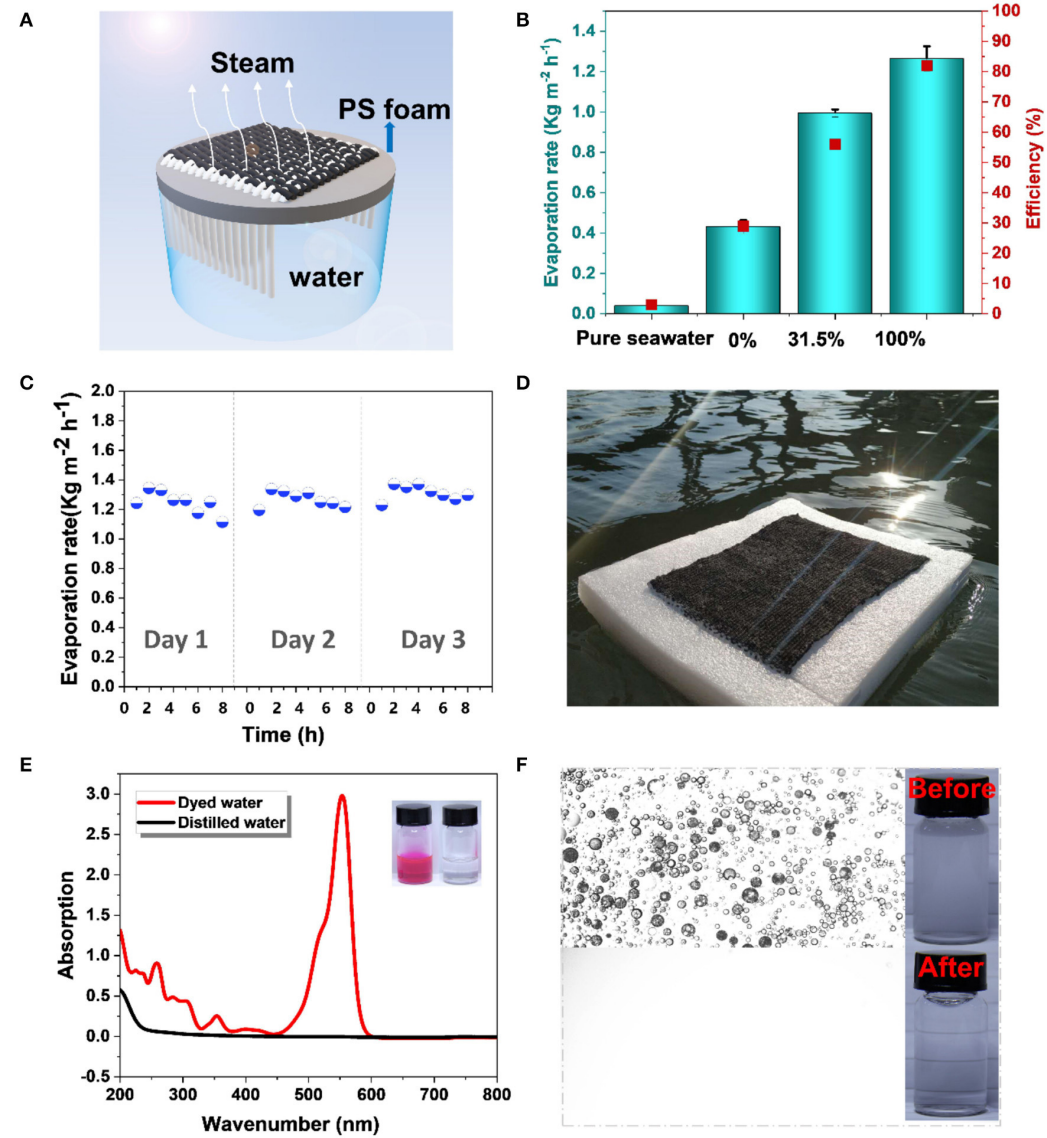
**(A)** The scheme of an interfacial solar generator; **(B)** The evaporation rates and conversion efficiency of the simulated seawater without a generator and with generators of different values of CBS coverage at one sun illumination; **(C)** The evaporation rates of the simulated seawater with a 100% CBS coverage generator for 3 days at one sun illumination; **(D)** A large-scale 100% CBS coverage generator on the sea; **(E)** UV-vis absorption spectra and photos of dyed water and distilled water; and **(F)** Optical microscopic images and photos of oil-in-water emulsion before and after the purification (one sun).

Figure 5A shows the design of a solar steam generator. Now, cotton yarns were involved because they are hydrophilic and can promote the water transfer from the reservoir (underlying water) to the heated fabric surface. The top layer is a photothermal sensitive and hydrophilic CBS/cotton fabric. The bottom layer is a PS foam, which allows the devices to float on the water surface. As indicated in Figure 5B, with a gradual increase in the CBS coverage from 0 to 100%, the CBS/cotton solar steam generators show a significant increase of the evaporation rate of 0.039, 0.431, 0.990, and 1.250 kg m^−2^ h^−1^, respectively. The highest evaporation rate was more than 31 times higher than the rate of water evaporation in the natural environment. The water evaporation rate was tested in a 60-min test period (Supplementary Figure 5). The corresponding evaporation efficiency was further calculated by using different temperatures recorded by an IR thermal imaging device. The surface temperature of a generator with 100% CBS coverage rapidly increases from 24.5 to 41.5°C under one sun illumination (Supplementary Figure 6). The temperature of the aqueous salt solution without a generator remains almost the same under some test conditions. In our measurements, the best conversion efficiency of a solar steam generator reached 82%, which is around 1.03–1.29 times higher than that of the other solar steam generators (Hua et al., 2017; Chen et al., 2018).

As interfacial solar steam generators have been proven to be an energy-saving and efficient technique for sewage treatment and desalination, we then tested the performance of the CBS/cotton solar steam generator in these application scenarios. To estimate the desalination performance, aqueous salt solution (3.5 wt% KCl) was used to simulate seawater, and the CBS/cotton solar steam generators with an area of 4 × 4 cm were applied to evaporate this solution. For this CSB/cotton generator, 2 ml of pure water per hour was obtained from the simulated seawater of 250 ml at 1 sun illumination. In addition, CBS/cotton solar steam generators exhibit their advantages in self-refreshability and recyclability during long-term use. After desalination for 8 h, a large number of salt crystals are precipitated on the upper surface layer of the CBS/cotton fabric (Supplementary Figure 7), and the crystallization of the salts increases with time. Although the evaporation rate is slightly decreased due to the accumulation of salt crystals on the surface (Figure 5C), those salts generated by the daylight can be fully redissolved into the solution at night (Supplementary Figure 7). The porous structure in a fabric provided a plenty of water channels to allow a thoroughly redissolving and mass transfer when the evaporation process slowed down at night. As a result, the evaporation rate of the CBS/cotton solar steam generator can be recovered on the second day (Figure 5C). Such a feature ensured the long-term stability of the CBS/cotton solar steam generator for long-term operation. It should be noted that although a 4 × 4 cm generator was used in these quantitative tests, we can obtain a structure of any size and can produce a larger size generator through manual or machine weaving approaches. The amount of water that can be evaporated is positively related to the surface area of a generator. For example, a 20 × 20 cm generator is expected to evaporate 0.4 kg of water during 8 h at one sun illumination (Figure 5D).

To assess the performance of the CBS/cotton solar steam generator for sewage treatment, two simulated pollutants, including dye solution (200 ppm Rhodamine-B aqueous solution) and oil-in-water emulsion (200 ppm), were also used for evaporation. The corresponding evaporation process was recorded in Movie 2. For a generator with 100% CBS coverage, the evaporation rates of Rhodamine-B solution and oil-in-water emulsions are 1.36 and 2.00 kg m^−2^ h^−1^, respectively. These values are 40 times higher than those of the solution system without a generator (Supplementary Figure 8). The water collected from the evaporation of Rhodamine-B solutions is transparent with the purity of 99.99% (Figure 5E). Clean distilled water was also obtained by the evaporation of oil-in-water emulsion. No oil drops were detected in the evaporated water (Figure 5F). It should be noted that, in this work, the basic plain-weaving was applied with the CBS yarn as warp and the cotton yarn as weft, which created a relatively tight textile structure and an easy solution transportation from the cotton to the CBS yarn interface (Supplementary Figure 9). However, the surface area of the CBS in contact with air is relatively low in this tight textile structure. More precise textile structure designs are desired, considering the different functions of the two yarns, as well as the maintained solution transfer and improved evaporation efficiency. Since there is not much difference in the hardness and flexibility between the CBS yarn and cotton yarn, they can be easily operated with controlled proportions and structures by a commercial small-sized loom. By adjusting the density of the textile and the braiding form of the two yarns, lighter weight and higher efficiency might be achieved in the future work.

## Conclusions

In summary, we report a one-step scalable heating process to carbonize the animal silks. In such an approach, no additives and activation processes are required. The resultant carbonized silks are electroconductive (with conductivity up to 2,387 S cm^−1^), mechanically robust (mechanical strength can tolerate further yarn-spinning and weaving), and can provide high photothermal absorption efficiency (82%). Profit from these characteristics, we then assembled these carbonized silks into a fluidic electrogenerator and fabric-based solar steam generator and evaluated their functional performance. The fluidic electrogenerator, consisting of a CBS electrode (4 cm in length) and a copper wire electrode (4 cm in length), can output a voltage of 0.35 V under the condition of simulating the sea environment. On the other hand, the CBS/cotton solar steam generator provided a useful water purification function with an evaporation rate of 1.25, 1.36, and 2.0 kg m^2^ h^−1^ for simulated seawater, dyed solution, and oil-in-water emulsion, respectively. This work is expected to guide a large-scale preparation and use of animal silk-derived amorphous carbon fibers.

## Data Availability Statement

The original contributions generated for the study are included in the article/Supplementary Material, further inquiries can be directed to the corresponding author/s.

## Author Contributions

All authors listed have made a substantial, direct and intellectual contribution to the work, and approved it for publication.

## Conflict of Interest

The authors declare that the research was conducted in the absence of any commercial or financial relationships that could be construed as a potential conflict of interest. The handling Editor declared a past co-authorship with one of the authors SL.
