# Supplementary material for: Animal Silk-Derived Amorphous Carbon Fibers for Electricity Generation and Solar Steam Evaporation
Source: Front Chem. 2021 Jun 22;9:669797. doi: 10.3389/fchem.2021.669797 (PMC8259506; doi:10.3389/fchem.2021.669797)
Supplement: Supplementary file 1 [file Data_Sheet_1.docx]

Supporting Information

Animal silks derived amorphous carbon fibers for electricity generation and solar steam evaporation

*Ping Qi*^§^*, Jing Ren*^§^*, and Shengjie Ling*^§,*^

^§^ School of Physical Science and Technology, ShanghaiTech University, 393 Middle Huaxia Road, Shanghai 201210, People’s Republic of China


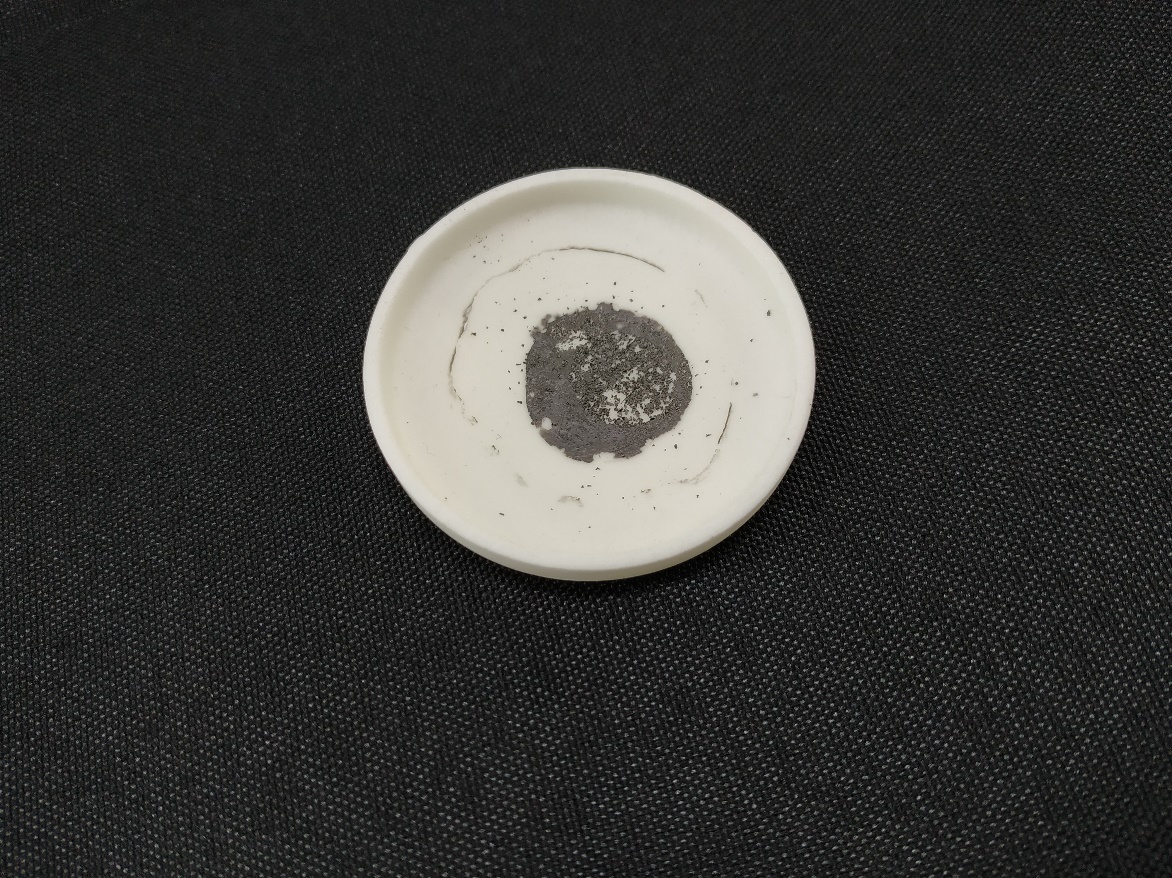


**PA66**


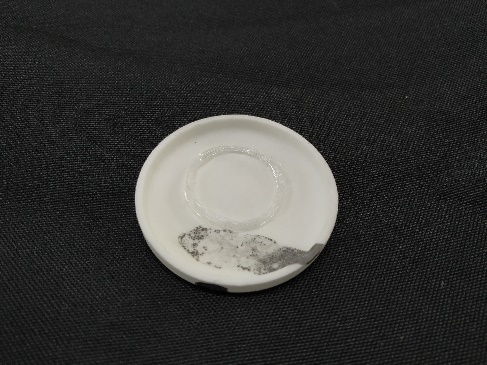


**2 cm**

carbonization


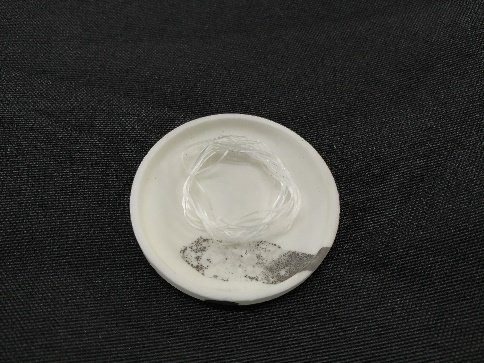

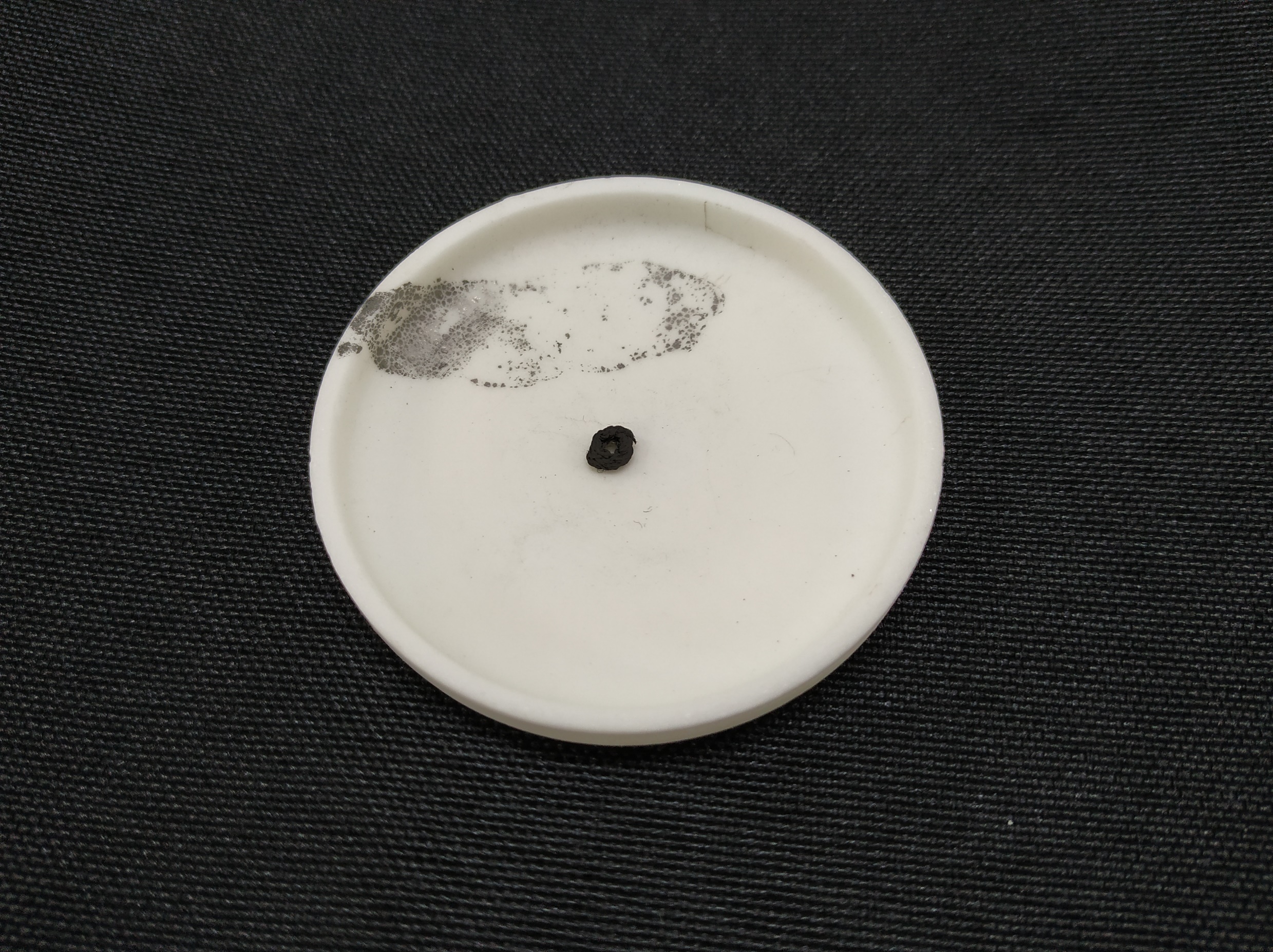


**2 cm**

**PAN**

carbonization

**1 cm**

Figure S1. PA66 and PAN fibers before and after the carbonization process (1000℃).


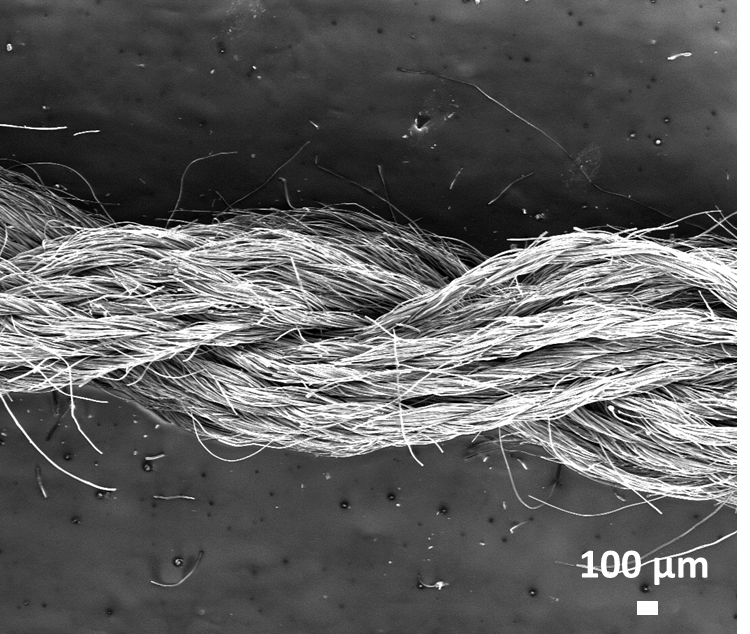


Figure S2. The morphology of a CBS yarn (carbonized at 1000℃).


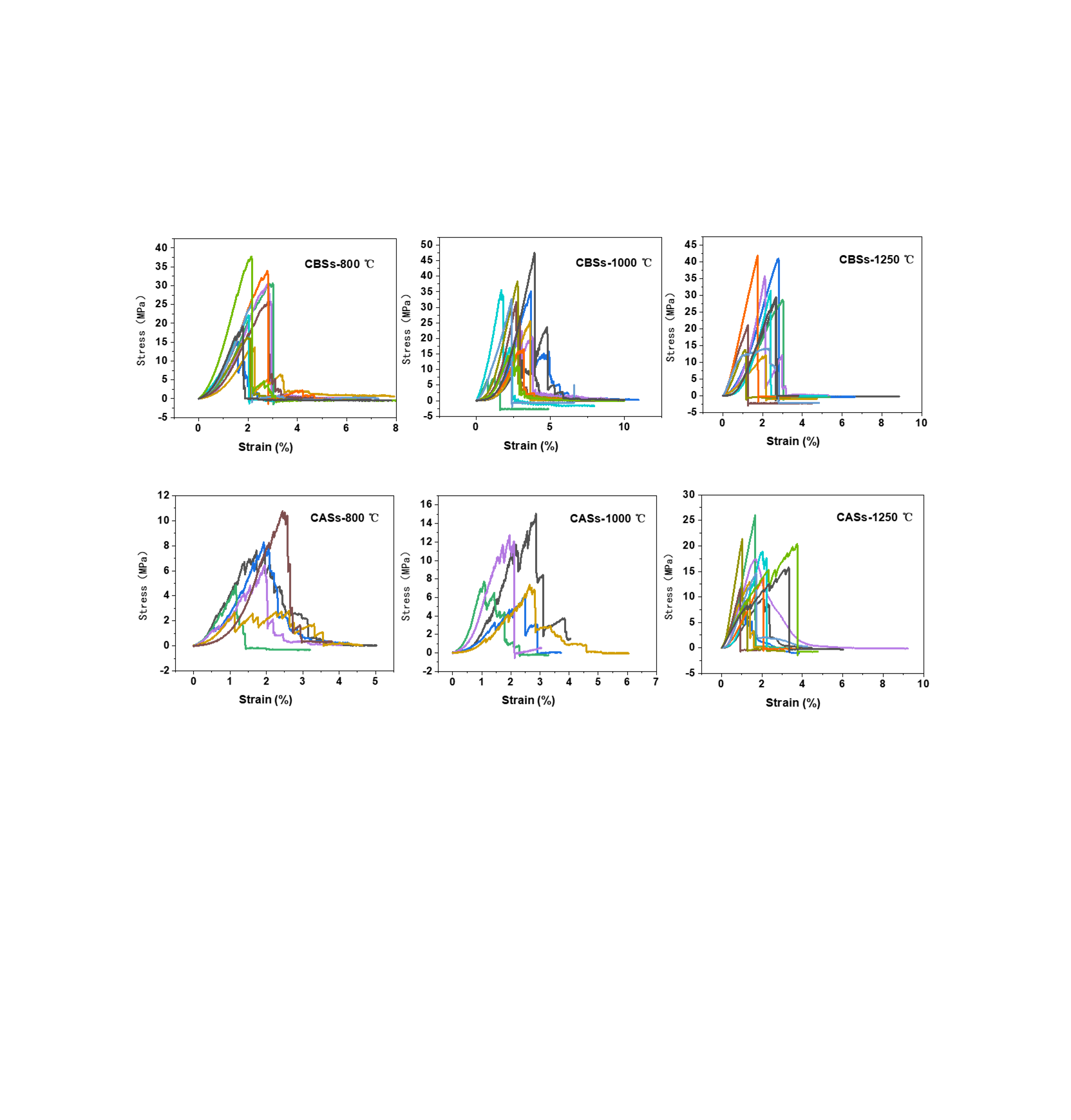


Figure S3. The mechanical properties of CBSs (top) and CASs (bottom).


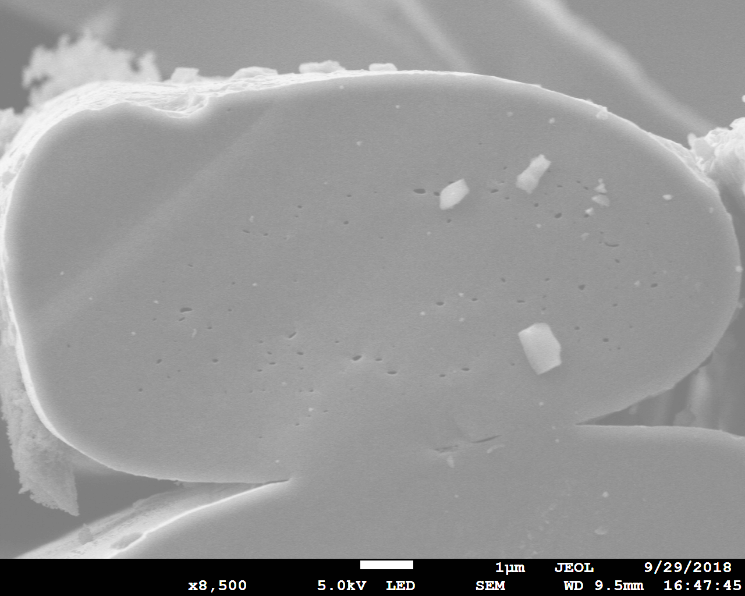

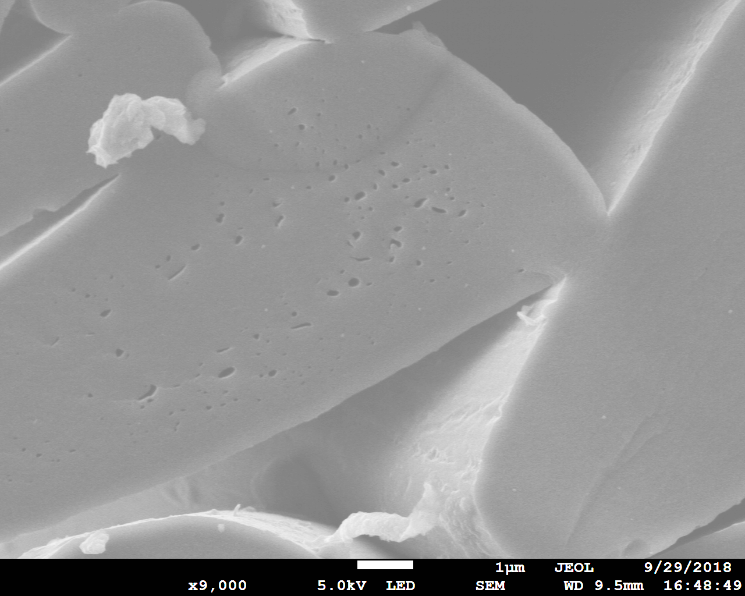

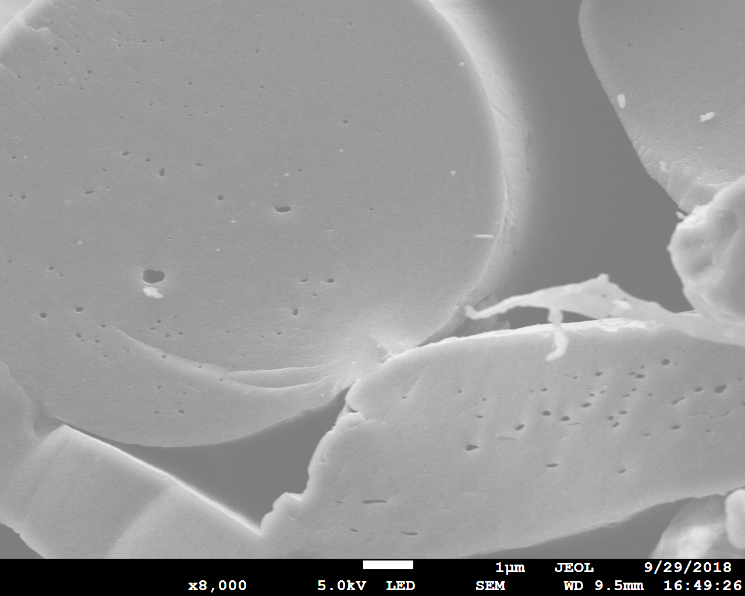


Figure S4. The cross-sectional SEM images of CASs (carbonized at 1000℃).


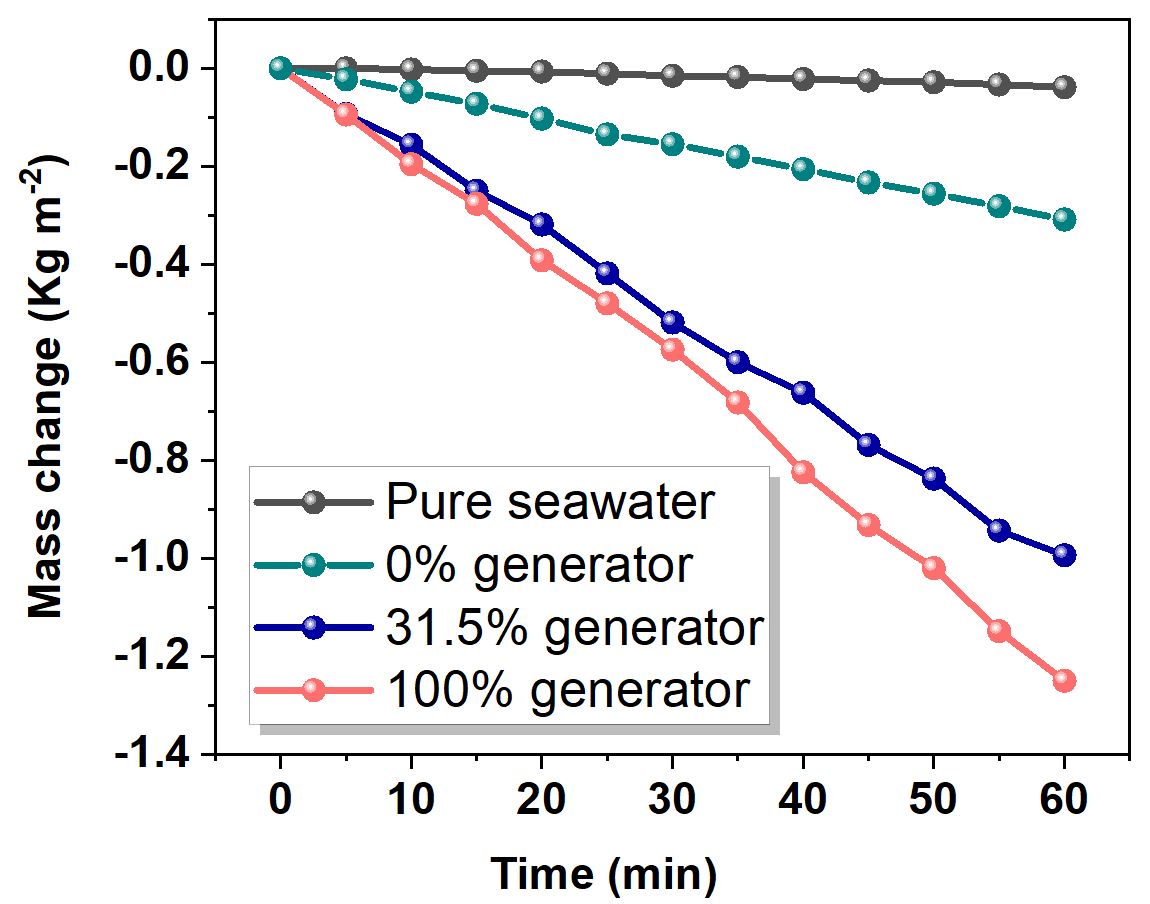


Figure S5. Water evaporation rates tested every five minutes in a 60-minute test period.

Figure S6. The temperature change on the surface of a 100% CBS coverage generator.


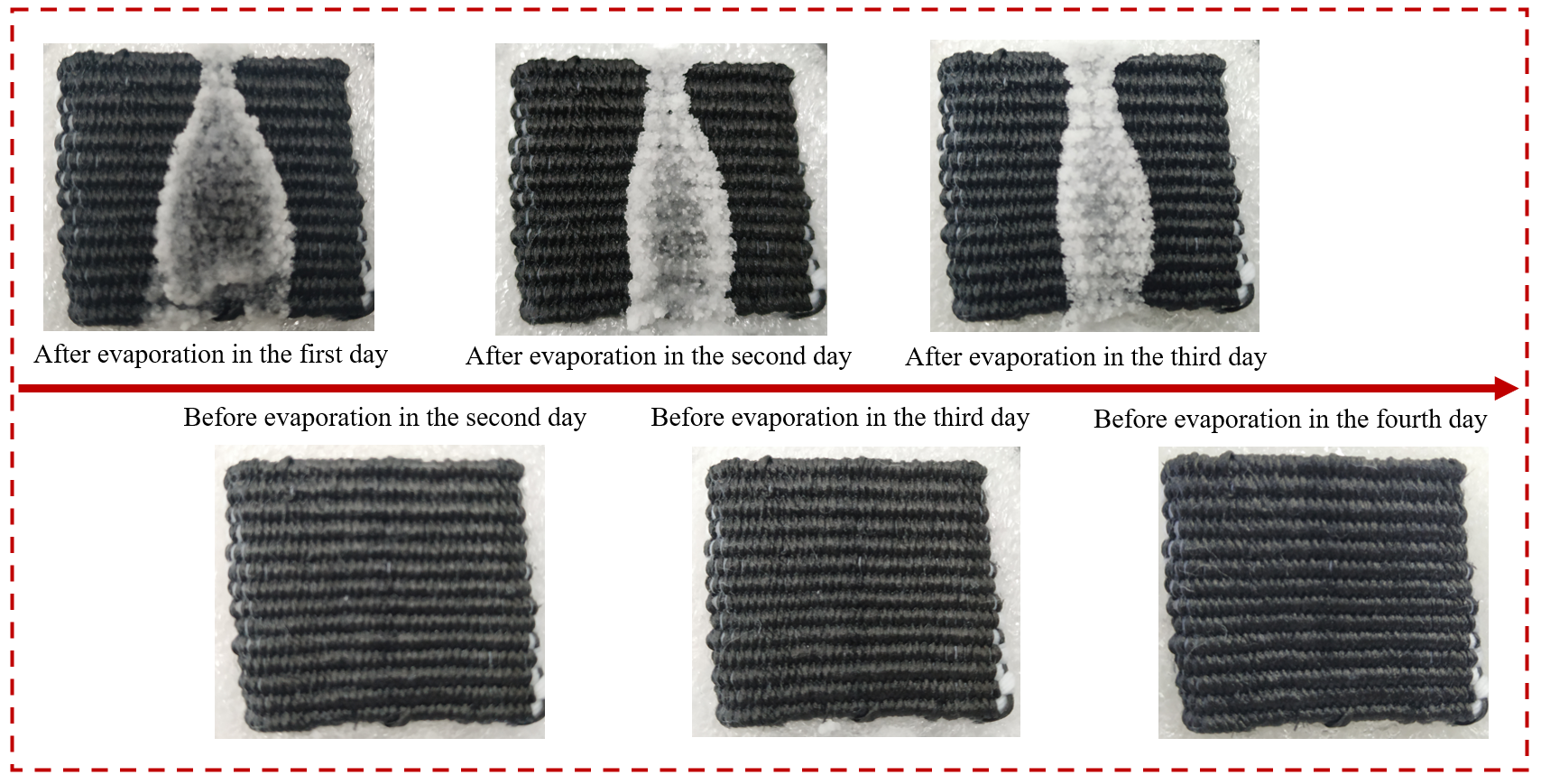


Figure S7. Salts precipitated from saline solution after evaporation for 8 hours (top) in the first, second or third day respectively; salts dissolved in saline solution at night (bottom).


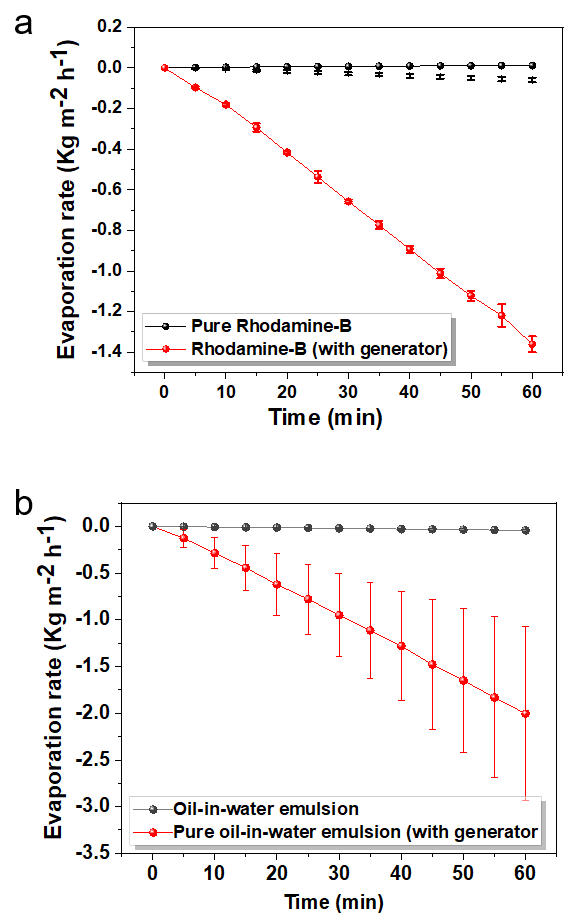


Figure S8. The evaporation rates of Rhodamine-B solution (a) and oil-in-water emulsions (b) with and without a generator.


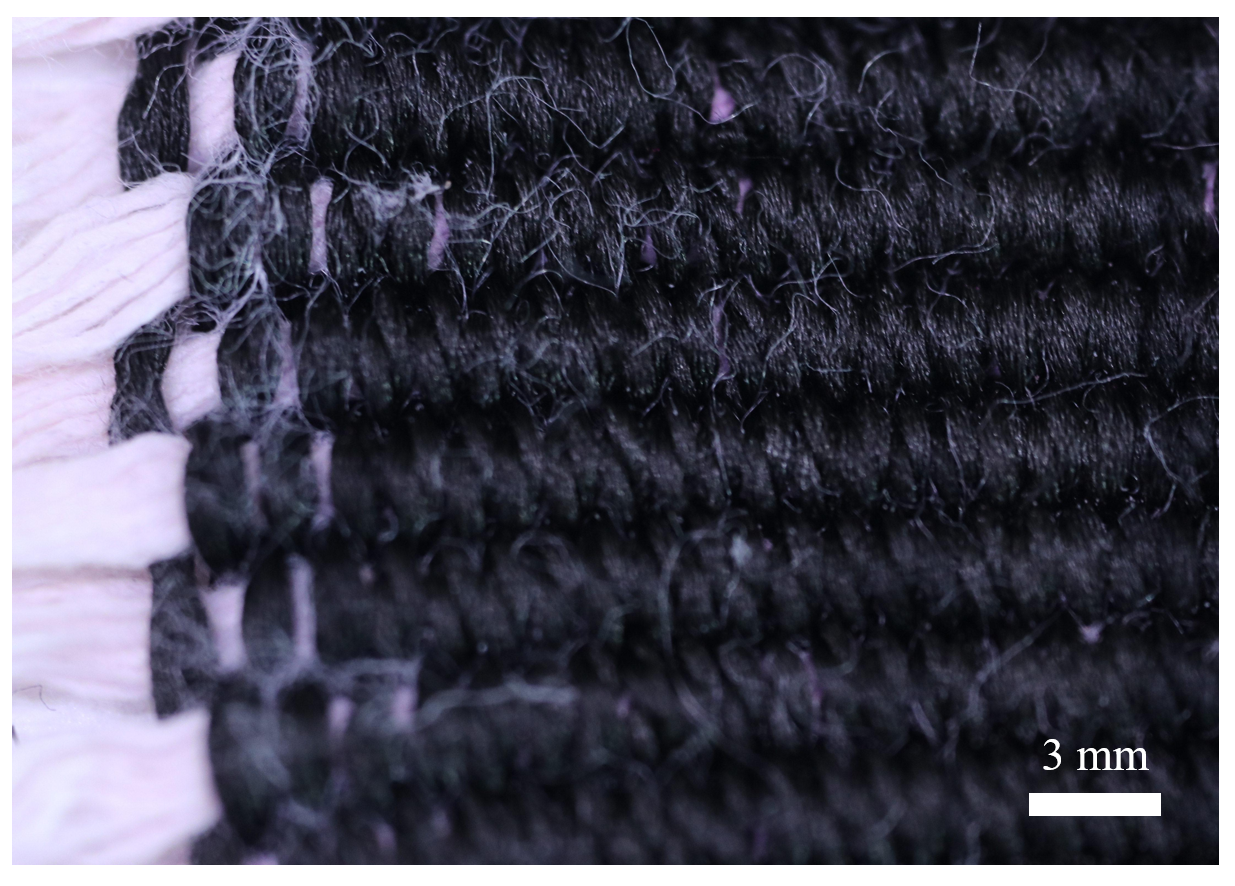


Figure S9. Image of a composite fabric made by CBS yarns and cotton yarns.Table S1. Electrical conductivity of CBSs

| Carbonization temperature (℃) | Electrical conductivity（S cm^-1^） |
| --- | --- |
| 800 | 18 |
| 1000 | 322 |
| 1250 | 2387 |

Table S2. The mechanical parameters of CBSs and CASs.

| Silk fibers | Carbonization  Temperature (℃) | Modulus  (GPa) | Strength  (MPa) | Strain  (%) | Toughness  (MJ/m^3) |
| --- | --- | --- | --- | --- | --- |
| CBSs | 800 | 1.2±0.2 | 24±8 | 3±1 | 27±10 |
|  | 1000 | 1.2±0.3 | 27±10 | 3±1 | 37±10 |
|  | 1250 | 1.5±0.6 | 30±13 | 2±1 | 26±20 |
| CASs | 800 | 0.4±0.2 | 7±2 | 3±1 | 8±3 |
|  | 1000 | 0.4±0.3 | 10±4 | 3±1 | 12±6 |
|  | 1250 | 1.0±0.5 | 16±5 | 2±1 | 18±11 |

Table S3. The measured diameters of voids of CASs.

| Diameter of Voids | CASs-1 | CASs-2 | CASs-3 | Average |
| --- | --- | --- | --- | --- |
| Small (μm) | 0.083 | 0.021 | 0.027 | 0.044 |
| Large (μm) | 0.212 | 0.066 | 0.098 | 0.125 |
